# First case of *Trichinella nativa* infection in wild boar in Central Europe—molecular characterization of the parasite

**DOI:** 10.1007/s00436-017-5446-6

**Published:** 2017-04-25

**Authors:** Ewa Bilska-Zając, Mirosław Różycki, Ewa Chmurzyńska, Ewelina Antolak, Marek Próchniak, Katarzyna Grądziel-Krukowska, Jacek Karamon, Jacek Sroka, Jolanta Zdybel, Tomasz Cencek

**Affiliations:** National Veterinary Research Institute in Puławy, Partyzantów Avenue 57, 24-100 Puławy, Poland

**Keywords:** *Trichinella nativa*, Wild boars, Haplotype, 5s rDNA-ISR, CO1

## Abstract

The examination of wild boars gained in Poland shows for the first time occurrence of *Trichinella nativa*, freeze-resistant species of *Trichinella* in this host from the central Europe region. This finding is not only one of several cases of *T. nativa* invasion in wild boars all over the world but also one of the very few cases of *T. nativa* detected so far beyond the known boundary of occurrence of this species. The molecular characterization of discovered larvae based on analysis of partial genes: 5s rDNA-ISR and CO1 confirm the findings. Moreover, the analyzed DNA sequences of both genes present new haplotypes of *T. nativa* in comparison to that described previously.

## Introduction

Nematodes of the genus *Trichinella* are zoonotic parasites with a high impact on human health and animal trade (Gottstein et al. 2009). Till now, 12 taxa in this genus are recognized and divided into two clades: encapsulated and non-encapsulated ones. From these 12 known taxa, 4 are circulating in Europe, 3 encapsulated (*Trichinella spiralis*, *Trichinella nativa*, *Trichinella britovi*) and one non-encapsulated (*Trichinella pseudospiralis*) (Pozio and Zarlenga 2013). All of them are infective to human and consumption of meat with live larvae poses a risk of trichinellosis for humans.

Individual species differ by their properties and zoonotic potential. These characters influence the risk assessment in humans and may affect the development of new sanitary and veterinary regulations. For example, discovery of the *T. pseudospiralis* in wild boars in Poland (Bilska-Zajac et al. 2016) resulted in the limitation of the compressor method use in endemic regions. Information on *Trichinella* species occurring in the country is also important for veterinary services of third countries in the supervision of imports of food of animal origin. Therefore, compulsory examination for trichinellosis in accordance with Commission Implementing Regulation (EU) 2015/1375 of 10 August 2015 laying down specific rules on official controls for *Trichinella* in meat, has two phases and includes meat inspection to detect larvae and, in the case of positive results, identification of *Trichinella* species causing the invasion. In Poland routine inspection (detection of parasites) of meat from pigs, wild boars, coypus, and horses is provided by veterinary inspection services. The National Veterinary Research Institute as a national reference laboratory for trichinellosis in Poland is responsible for confirmation of positive results and species identification. From four routinely examined animal species, trichinellosis is discovered the most often in wild boars. It should be stressed that in the last decade, human trichinellosis was caused by ingestion of meat from illegally hunted and not-examined *Trichinella*-infected wild boars only.

It should be taken into consideration that the wild boar population in Poland increased from the last two decades from 90,000 up to 300,000 (Popczyk 2016). With the increase of the population, the number of *Trichinella*-infected animals is rising and now achieved over 600 positive animals per year. Up to 2012, only two species: *T. spiralis* and *T. britovi* were discovered in wild boars in Poland (Bilska-Zając et al. 2013). Since 2012, a few cases of *T. pseudospiralis* were also detected (Bilska-Zajac et al. 2016). However, to date, *T. nativa* discovered in few Northeastern European countries, was never detected in wild boars in Poland. Until now, this species was detected in Poland only once in red fox (Chmurzynska et al. 2013).

From January to September 2016, 300 muscle samples from wild boars previously recognized by VIS laboratories as *Trichinella* positive were delivered to NVRI for species identification. The species identification was done by multiplex PCR according to the protocol of European Reference Laboratory for Parasites. Altogether, 235 samples were identified as *T. spiralis* (78.4%), 57 samples as *T. britovi* (19%), and in 7 samples the mixed infection *T. spiralis/T. britovi* was recognized (2.3%). One sample was identified as *T. nativa* (0.3%). The sample of *T. nativa* was originated from wild boar gained in Ławice, district Iława, Varmian Masurian province. The infected wild boar was female, close to 2 years old, and weight of carcass after removing of the guts were about 40 kg. Intensity of invasion was evaluated as one larvae per 10 g of muscle tissue. Because this was the first finding of *T. nativa* in wild boar in Poland (and Central Europe) we aimed to perform the genetic analysis of isolated larvae.

## Material and methods

For the study, five larvae extracted from the muscle of the positive wild boar were used. The DNA from each larvae were separately extracted using commercial kit according to manufacturer protocol (Promega, USA). The recognition of species were provide by multiplex PCR method according to European Reference Laboratory method (Zarlenga et al. 1999).The PCR products were separated electrophoretically in 1.5% agarose gel and stained with Simply Safe (EURx, Polska). DNA bands in gel were visualized under UV light. For every PCR, negative (nuclease-free water) and positive (reference *Trichinella* larvae ISS3, ISS2, or ISS13 from EURLP) controls were used.

Subsequently, DNA of each *T. nativa* larvae was designated for molecular characterization. The part of 5S ribosomal DNA intergenic spacer region (5s rDNA-ISR) and fragment of mitochondrial cytochrome c oxidase 1 (CO1) gene were amplified for confirmation of species identification and molecular characterization of detected *T. nativa*. For amplification of 5s rDNA-ISR, forward primer 5′-GCGAATTCTTGGATCGGAGACGGCCTG and reverse primer 5′-GCTCTAGACGAGATGTCGTGCTTTCAACG as described earlier by Liu et al. (1996) and Rombout et al. (2001b) were used (Liu et al. 1996; Rombout et al. 2001a). The expected PCR products were approximately 750 base pairs. A second PCR was performed to amplify the CO1 gene, using forward primer 5′-TACCTATACTACTAAGAGGATTCGGA and reverse primer 5′-CTAGTACTCATAGTATGGCTGGTG previously designed by Franssen et al. (2015). The amplification results in products of approximately 760 bp. For amplification of both 5s rDNA-ISR and CO1 gene, the same condition and mix reagents were used as described below. Polymerase chain reactions were performed in 50-μL volume containing 4 μL of extracted DNA, 30 μL of GoTaq G2 Master Mix (Promega, USA), and 0.5 μL of each primers (10 mM). The PCR conditions were as follows: denaturation at 95 °C for 15 min followed by 40 cycles of 95 °C for 1 min, 50 °C for 1 min, 72 °C for 1 min and 30 s, and a final elongation step of 72 °C for 10 min. The PCR products were separated elecrophoretically in 2% agarose gel with Simply Safe dye. DNA bands in gel were visualized under UV light. PCR products were purified using ExoSAP (Affymetrix, UK) according to manufacturer procedure and sent for sequencing to Genomed-sequence company.

Sequence and phylogeny analysis were done using available computer software. Forward and reverse sequences were aligned and edited manually using the Geneious R7 (Kearse et al. 2012). The consensus sequences of 5s rDNA-ISR were applied for alignment. Moreover, for the analysis, 5s rDNA-ISR sequences of *T. spiralis* (GenBank Accessions KJ716745, AY009946); *T. britovi* (GenBank Accession AY009943, GU325734, GU325737); *T. nativa* (GenBank Accession AY009944); *T. murelli* (GenBank Accession AB426627); T6 (GenBank Accession AY009948); T8 (GenBank Accession AY009949); T9 (GenBank Accession KP900345); and *T. pseudospiralis* (GenBank Accession AY009943) were used.

**Fig. 1:**
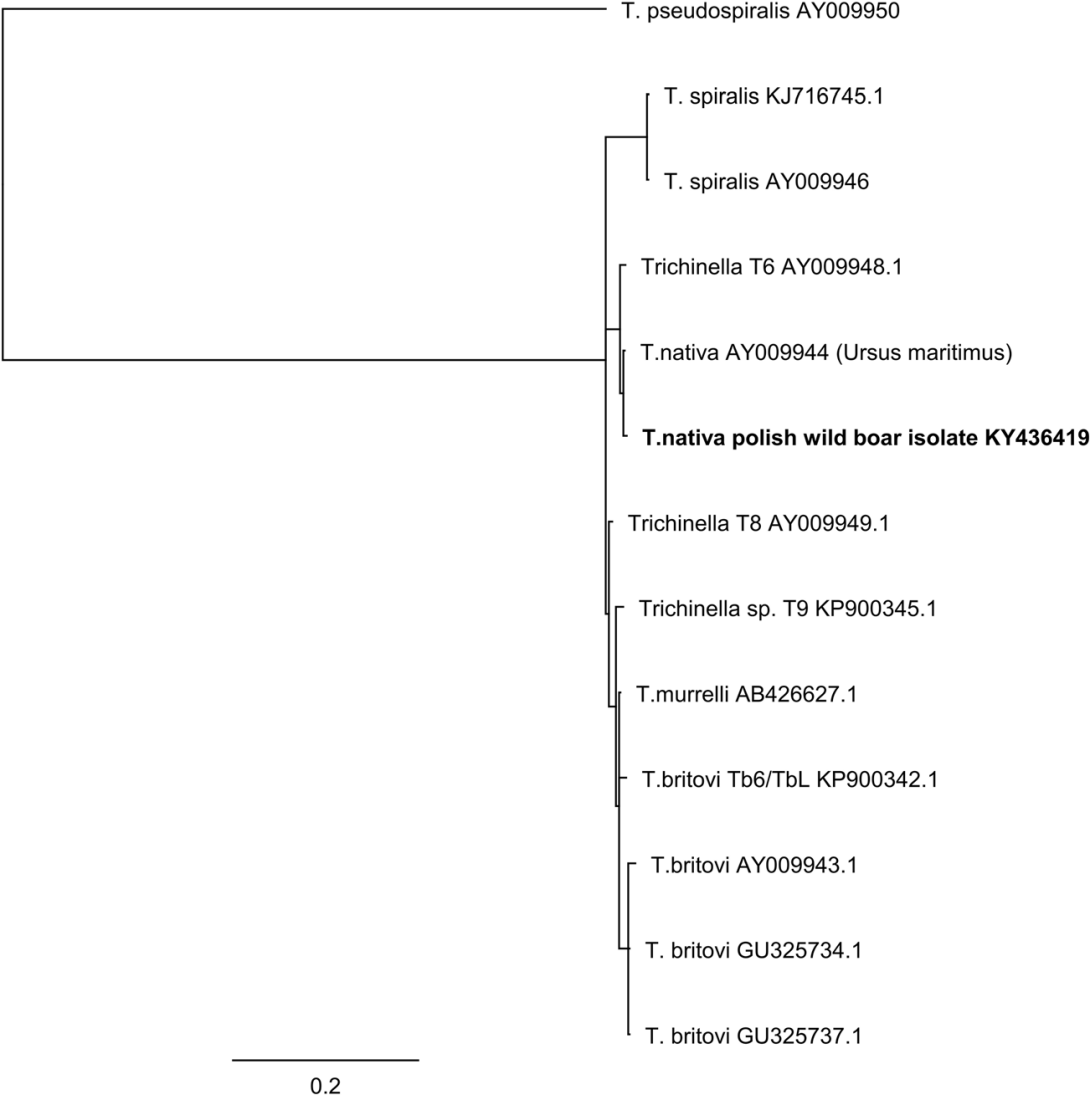
Phylogenetic tree of the partial of 5s rDNA-ISR gene sequences from the *Trichinella* larvae. The phylogenetic tree was constructed using MrBayes analyses running the HKY model with burn in  =  100,000. The tree constructed using the Neighbor-Joining method with Jukes-Cantor model shows identical results, data not shown

**Fig. 2.**
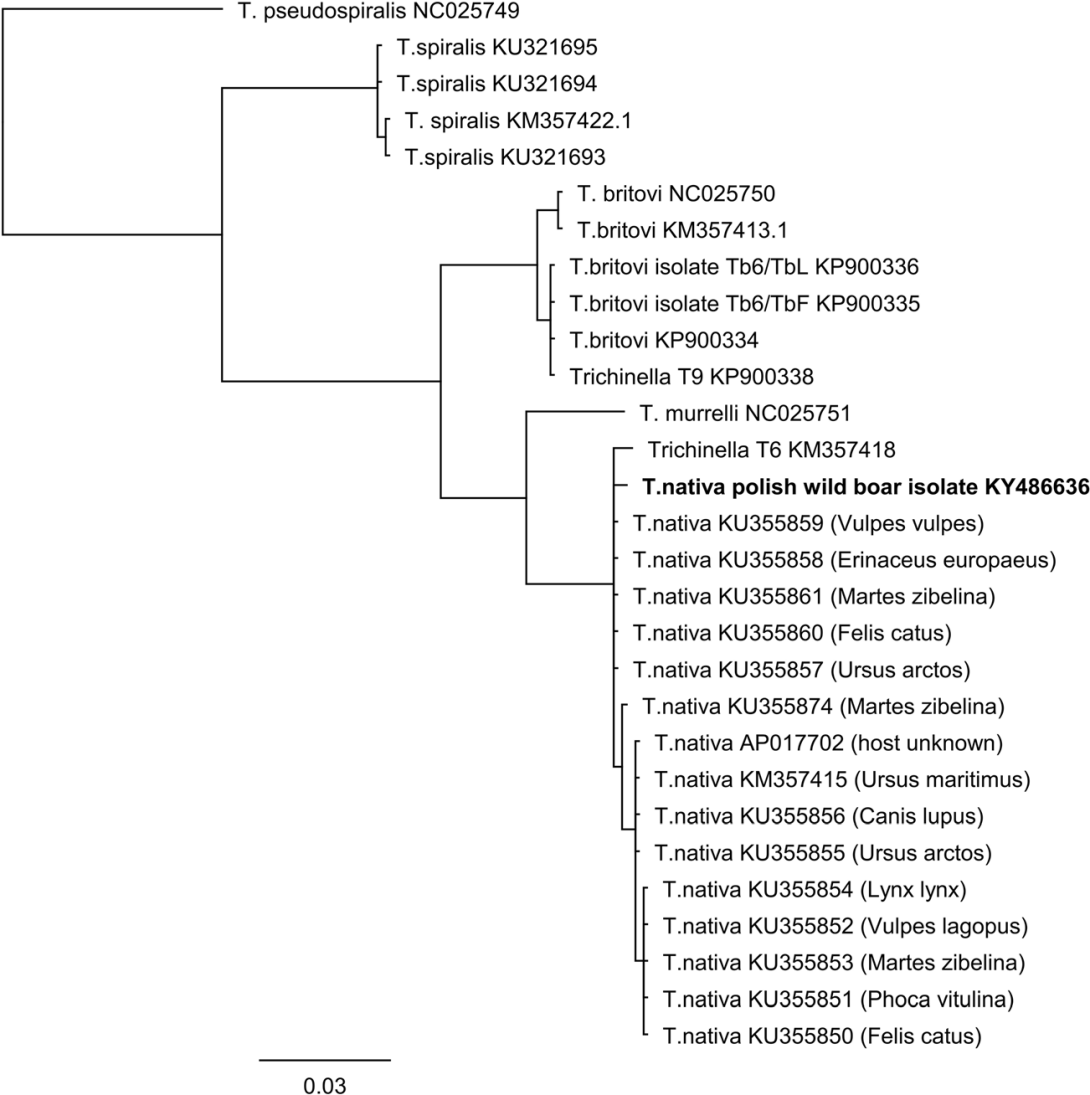
Phylogenetic tree of the partial of CO1 gene sequences from the *Trichinella* larvae. The phylogenetic tree was constructed using MrBayes analyses running the HKY model with burn in  =  100,000. The tree constructed using the Neighbor-Joining method with Jukes-Cantor model shows identical results, data not shown.

Analogical analysis was performed using the consensus of mitochondrial CO1 sequences. For the alignment the CO1 sequences of *T. spiralis* (GenBank Accessions KM357422, KU321693, KU321694, KU321695); *T. britovi* (GenBank Accessions KP900334, KP900335, KP900336, NC025750, KM377413); *T. nativa* (GenBank Accessions KU375850–KU375861, KU375874, KM357415, AP017702); *T. murelli* (GenBank Accession NC025751); T6 (GenBank Accession KM357418); T9 (GenBank Accession KP900338) and *T. pseudospiralis* (GenBank Accession NC025749) were used. Subsequently, phylogenetic analysis of consensus sequences was performed using the Neighbor-Joining method with Jukes-Cantor model and MrByes method in Geneious R7 (Figs. 1, 2).

## Results

The amplification of DNA from investigated larvae resulted in multiplex PCR in band 127 bp for all five larvae. The result indicate on singular infection of *T. nativa*. Subsequently, the amplification of chosen for molecular analysis 5s rDNA-ISR and CO1 genes resulted in products 750 and 760 bp, respectively.

The sequencing of 5s rDNA-ISR amplicons resulted in obtaining sequences from all five individual larvae. The total length of comparative alignment studied in this research was 730 bp. The obtained sequences of 5s rDNA-ISR from all five individual muscle larvae had 100% identical haplotype. Nucleotide sequence data obtained in the study were deposited in GenBank under the following accession number: KY436419. The total length of alignment of our *T. nativa* 5s rDNA-ISR sequences with sequences from GenBank database was determine by length of shortest entry and were 463 bp length. The phylogenetic analysis of obtained alignment from 5s rDNA-ISR sequences confirmed species identification of detected larvae as *T. nativa*. Furthermore, according to the generated phylogenetic tree, the analyzed sequences were clustered in separated clad with *T. nativa* and T6 (Fig. 1).

Sequence analysis of all five obtained 5s rDNA-ISR sequences shows 99.8% of similarity with *T. nativa* AY009944, differing in one single nucleotide polymorphism in the position 136 (Table 1). The matrix identity analysis shows 99.1% identity with *Trichinella* T6 AY009948 with three SNP differences in position 93, 136, and 391 (Table 1).

**Table 1:** Position of single nucleotide polymorphism between *T. nativa* 5S rDNA-ISR haplotype generated in this study and sequences of other *Trichinella* species and genotypes

Nucleotide position/ *Trichinella* species	93	136	391
*T. nativa* polish isolate KY436419	T	*G*	A
*T. nativa* AY009944	T	C	A
T6 AY009948	C	C	G
*T. spiralis* KJ716745, AY009946	T	T	G
*T. britovi* KP900342, AY009943, GU325734, GU325737	T	G/C	G
*T. murelli* AB426627	T	C	G
T9 KP900345	T	C	G
*T. pseudospiralis* AY009950	T	C	G
T6 AY009948	T	G	A

The sequencing of a part of CO1 gene resulted in obtaining sequences from all five individual larvae. The total length of comparative alignment studied in this research was 750 bp. The obtained sequences of CO1 from all five individual muscle larvae had 100% identical haplotype. Nucleotide sequence data obtained in the study were deposited in GenBank under following accession number KY486636. The total length of alignment of our *T. nativa* CO1 sequences with *Trichinella* sequences from GenBank database was determined by length of shortest entry and were 721-bp length. Phylogenetic analysis of obtained alignment from partial CO1 sequences also confirmed species identification of analyzed larvae. The obtained sequences of first detected in Poland *T. nativa* were clustered in one clad with others *T. nativa* chosen to analysis. The resulting clad was consist from five groups of *T. nativa* haplotypes, one of the group were created only from newly detected *T. nativa* (Fig. 2). Sequence analysis of all five obtained CO1 sequences show the highest 99.7% identity with *T. nativa* KU355857, KU355858, KU355859, KU355860, and KU355861. The detailed analysis of matrix identity shows from 99.2 to 99.7% identity with various *T. nativa* CO1 sequences and 99.2% identity with *Trichinella* T6. The investigated CO1 sequence differed between vary sequences of *T. nativa* deposited in GenBank from two to six single nucleotide polymorphisms (Table 2).

**Table 2:** Position of single nucleotide polymorphism between *T. nativa* CO1 haplotype generated in this study and sequences of other *Trichinella* species and genotypes

Nucleotide position/ *Trichinella* species	98	129	144	438	564	624	626	639
*T. nativa* polish isolate KY486636	*C*	T	*A*	T	C	T	C	T
*T. nativa* KU355853 *T. nativa* KU355850, *T. nativa* KU355851, *T. nativa* KU355854	T	C	G	T	T	C	C	C
*T. nativa* KU355857 *T. nativa* KU355858, *T. nativa* KU355859, *T. nativa* KU355860 *T. nativa* KU355861	T	T	G	T	C	T	C	T
*T. nativa* KU355855 *T. nativa* KU355856 *T. nativa* KM357415	T	T	G	T	C	C	C	T
*T. nativa* DQ007891	–	–	–	–	–	C	C	T
*T.nativa* AF129489	–	–	–	–	–	T	C	T
*T. nativa* AP017702	–	–	–	–	–	T	C	T
*T. nativa* AB252966	–	–	–	–	–	T	C	C
*T. nativa* KU355874	T	C	G	T	C	T	C	T
T6 KM357418	T	T	G	C	T	T	T	T
*T. spiralis* KU321694, KU321695, KM357422.1, KU321693	T	T	A	C	T	C	T	C
*T. britovi* NC025750, KM357413, KP900336, KP900335, KP900334	T	T	A	T	C	T	C	T
*T. murelli* NC025751	T	T	A	T	T	T	C	C
T9 KP900338	T	T	A	T	C	T	C	T
*T. pseudospiralis* NC025749	T	T	A	C	T	T	C	C

## Discussion

In this study, we detected for the first time *T. nativa* in wild boar in Poland. This finding is not only one of several cases of *T. nativa* invasion in wild boars all over the world but also one of the very few cases of *T. nativa* detected so far beyond the known boundary of occurrence of this species. The natural geographic distribution of this cold-resistant species are arctic and subarctic areas of Holarctic regions. The border of the southern distribution of *T. nativa* has been identified between the isotherms −5 to −4 °C in January (Pozio et al. 1992; Pozio and La Rosa 2000; Pozio and Murrell 2006). In Europe, the species was detected mostly in Finland, Sweden, Russia, Estonia, Norway, Latvia, and Lithuania (Pozio 2016). The infected wild boar, described herein, was gained on the longitude and latitude 53° 34′ 50″ N, 19° 39′ 13″ E. The closest to our finding wild boar infected by *T. nativa* were detected in Lithuania, more than 500 km far from this outcome (https://www.iss.it, 2017.02.07). Our outcome is the first wild boar infected by *T. nativa* furthest to the South compared to the evidenced previously. Although occasional occurrence of this species below the known boundary were described, those cases were detected from other hosts. For example, findings of red foxes infected by this species in areas of Poland and Germany. The *T. nativa* isolate from red fox discovered in Poland in 2013 originated 150 km north-east far from the case of investigated herein wild boar (Chmurzynska et al. 2013). In Germany, the two red foxes infected by *T. nativa* were found 700 and 1200 km far to the south of known boundary of occurrence of this cold-resistant species (Chmurzynska et al. 2013). These geographic localizations of wild boar described here confirm results of previous researchers that the area of occurrence of *T. nativa* is spread onto new regions and new host species (Pozio and La Rosa 2000; Chmurzynska et al. 2013; Kirjusina et al. 2015; Pozio 2016).

The occurrence of this case have to be highlighted also because this is the first wild boar found to be infected by *T. nativa* in Central Europe. This species is known as the typical subarctic species of parasite, most often detected from terrestrial and marine carnivores (Gottstein et al. 2009; Kirjusina et al. 2015; Pozio 2016; Deksne et al. 2016; Odoevskaya and S. E. S. 2016). The carnivores had been described as typical host for this taxa and parasite can stay infected in muscle tissue of these animals at least 20 years (Kumar et al. 1990). It has to be underlined that both domestic pigs and wild boars are not common hosts for *T. nativa* (Kapel and Gamble 2000; Kapel 2001; Nockler et al. 2005; Hill et al. 2009). The tenacity study of this species indicates that their survival time in frozen meat is incomparable longer to other *Trichinella* species, especially to *T. spiralis*. Presented data in several studies indicate that *T. nativa* can survive in −18 °C for 5 years in polar bear, 4 years in arctic fox, and 1.5 year in wolf muscles (Dies 1980; Kapel et al. 1999; Dick and E. P. 2001). Those animal species are considered as typical hosts for *T. nativa*. It has to be highlighted that the survival time in wild boar muscles had not been recognized. However, for the experimentally infected domestic pigs, the survival time in −6.6 °C was 106 h (Hill et al. 2009). So that we can cautiously hypothesize that the time of resistance of this species in the wild boars muscles could be similar. Up to now, only several cases of wild boars infected by these cold-resistant species were evidenced (Kirjusina et al. 2015; Pozio 2016). The database of European Reference Laboratory reach 518 records of *T. nativa* from all over the world. Most of the cases are originated from lynx, red foxes, wolfs, black bears, wolverines, polar bears, and marine mammals and only eight cases were isolated from wild boars muscles (https://www.iss.it, 2017.02.07).

As the cases of detection of *T. nativa* in wild boars are very seldom, the molecular characterization of the parasite strains isolated from this host is incomplete. The genetic data available in GenBank are very limited. Therefore, in this study, for molecular analysis most often amplified in genetic studies, CO1 gene was used. According to available literature and genetic data, it seems that CO1 gives more opportunities to find possible differences between *T. nativa* strains compared to genes used in previous researches (cytB, 18s rDNA) (Odoevskaya and S. E. S. 2016). Additionally, in the study, we amplify and analyze also part of nuclear gene 5s rDNA-ISR. This gene was chosen especially for confirmation of species identification of multiplex PCR results. The database of both investigated parts of genes are not rich in sequences of *T. nativa*, especially from wild boars. Therefore, phylogenetic analysis was done on limited numbers of sequences from strains of *T. nativa* originated from different hosts excluding wild boars. The molecular analysis of 5s rDNA-ISR gene confirmed results of species identification done by multiplex PCR and showed that those detected in this study larvae belongs to the clad with other previously detected *T. nativa*. Unfortunately, the comparison of obtained sequences with those from GenBank was hampered by the shorter sequences, which caused excluding from alignment most of the deposited previous sequences. The highest identity with our 5s rDNA-ISR sequences shows *T. nativa* originated from polar bear (*Ursus maritimus*). The polar bear was detected in 1984 in Norway (Rombout et al. 2001b). The discovered one single nucleotide polymorphism between our sequences and this from polar bear indicate on occurrence of some genetic differences between *T. nativa* strains (Table 1). However, too less number of the 5s rDNA-ISR sequences of *T. nativa* in current study cause that further conclusions based on this analysis are impossible.

The phylogenetic analysis of CO1 sequences gives more robust comparison, because of evidence of higher number *T. nativa* sequences available in GenBank database and which were used in alignment. The analysis clustered our CO1 *T. nativa* sequences in one clad with the rest investigated *T. nativa* (Fig. 2). The indicated identity of our CO1 *T. nativa* sequences of 99.7 and 99.3% with sequences originated from Russia and Norway, confirm correct species identification but also show the differences between our sequences and these used in analysis. The discovered differences were shown in alignment, where several SNP’s between analyzed *T. nativa* sequences were detected (Table 2). Furthermore, two of SNP’s in position 98 (transition from T to C) and 144 (transition from G to A) were new substitutions comparing to the sequences previously deposited in GenBank, which means that this is a new *T. nativa* haplotype. However, it has to be highlighted that none of the CO1 sequences used in the study for alignment were from wild boars. The sequences from GenBank used for alignment originated from brown bears (*Ursus arctos*), lynx (*Lynx lynx*), seal (*Phoca vitulina*), stray cat (*Felis catus*), polar fox from fur farm (*Vulpes lagopus*), wolf (*Canis lupus*), hedgehog (*Erinaceus europaeus*), sables (*Martes zibelina*), and polar bear (*U. maritimus*). The analysis of these sequences do not show any significant connection of obtained haplotypes with host species. For example, in three sables three different haplotypes of CO1 sequences were found (Fig. 2). More probable is connection of CO1 *T. nativa* haplotypes with geographical origin of the hosts. The CO1 *T. nativa* sequences used in the analysis (besides our polish isolate) were originated in majority from Russia (13 sequences), Norway (1), and one with unknown origin. Our outcome confirm data presented by Odoevskaya and S. E. S. (2016), who reported several haplotypes of *T. nativa* discovered in various regions of Russia and concluded that haplotypes can be connected with the geographical origin of host. However, a small number of sequences which is noticed up to now and was used in the analysis limits possibilities to draw definitive conclusion.

The occurrence of several haplotypes of CO1 indicate on high genetic variation in this species of *Trichinella* nematodes. This could mean that *T. nativa* is a less homogeneous taxa compare to *T. spiralis* (Franssen et al. 2015; La Rosa et al. 2012) and may have higher amount of haplotypes, like for example, observed in *T. britovi* (Franssen et al. 2015). Furthermore, in the study observed, the high identity (99.2% in CO1 alignment) of investigated *T. nativa* sequences to T6 sequence. This could be explained by deriving both genotypes *T. nativa* and T6 from one linage (Korhonen et al. 2016).

## Conclusions

The first detection of *T. nativa* in wild boar in Poland is next evidence for the spread of this species on the new regions of Europe. This freeze-resistant species, rarely detected in wild boars, is infective for humans. *T. nativa* may cause danger for humans, who may acquire infection directly by consumption of meat of wild boar or indirectly by other infected vectors. Newly discovered haplotypes in current study (both, in 5 rDNA-ISR sec. and CO1 genes) are showing single-nucleotide differences compared with sequences deposited in GenBank and could indicate on high variation between *T. nativa* strains from various geographical region of host origin.
